# Communities need journals

**DOI:** 10.1098/rsnr.2016.0032

**Published:** 2016-08-31

**Authors:** Cameron Neylon

**Affiliations:** Centre for Culture and Technology, Faculty of Humanities, Curtin University, GPO Box U1987, Perth, WA 6845, Australia

What has changed for the scholarly journal over 350 years? What has remained the same? Many of our modern concerns, including the engagement of wider publics and the challenge to our academic conceptions of expertise, are not at all new, and many of the same issues were discussed at length in the seventeenth, eighteenth and nineteenth centuries. There are also moments of stark discontinuity. In the nineteenth century, dictionaries were published as periodicals; papers read at Royal Society meetings were refereed, but authors could normally make only ‘verbal’ (and not intellectual) changes to the text in response; the science writing of chemistry and physics was once categorized in the pages of some journals alongside poetry under the heading of ‘literature’.

If we are to learn from history then we need to hold ourselves to a high standard in trying to understand what it is (and is not) telling us. The stories we tell about the history of scholarly publishing are more frequently comfortable creation myths than evidence-based history.

So what has stayed the same? On one hand, the importance of groups and communities. On the other, the importance of authors, of professionalization and the distinctions created between communities of amateurs and of professionals. Together with the importance of language communities, of reading a paper (or having one's paper read) at the Royal Society and the development of journals as a means of creating research disciplines, I think this centrality of communities, of groups, of *clubs* is a strand that links us to the nineteenth century; and I think that is true because of the nature of knowledge itself.

Knowledge is a slippery concept, and I have made that argument elsewhere, so for now I will just assert that it belongs in the bottom right quadrant of Elinor Ostrom's categorization of goods (figure 1).^1^ Knowledge is non-rivalrous—if I give it to you I still have it—but also excludable—I can easily prevent you from having it by not telling you, or by locking it up behind a paywall or simply behind impenetrable jargon. This is interesting because James M. Buchanan's work on the economics of clubs shows us that it is precisely the goods in this quadrant that are used to sustain clubs and make them viable.^2^

**Figure 1.**
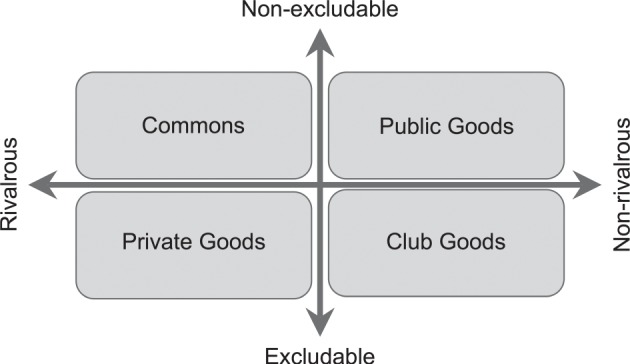
The typology of economic goods, after Ostrom (1990). The division of goods based on whether they are rivalrous and excludable creates four possible categories. Private goods, such as money or food, are rivalrous (if I give it to you I do not have it any more) and excludable. Public goods, such as air or access to justice, are (or at least should be) neither rivalrous nor excludable. Common pool resources, such as forests and fisheries, are difficult to exclude people from using, but are rivalrous (you cannot catch the same fish twice); whereas club goods are non-rivalrous, but can be retained as exclusive.

The survival of journals, or of scholarly societies, disciplines or communities, therefore depends on how they deploy knowledge as a *club good*. To achieve this deployment, it is necessary to make that knowledge, the club good, less exclusive and more public. What is nice about this view is that it allows us to talk about ‘public-making’ (Jan Velterop has used the old term ‘publicate’ in a similar way) as an activity which includes public engagement, translation and education, as well as scholarly publishing as we traditionally understand it, as overlapping subsets of this broader activity.

But what has changed? I would argue that the largest change in the twentieth century was one of scale. The massive increase in the scale and globalization of the research enterprise, as well as the rise of literacy, meant that traditional modes of coordination within scholarly societies and communities, and beyond to interested publics, were breaking down. Commercial players led by Robert Maxwell stepped in to address this coordination problem by bringing their industrial experience to what had been more like a cottage industry. We can frame this transaction as taking the club good, knowledge, and privatizing it by passing control of the content to this new class of industrial, commercialized publishers. As the form of this transaction solidified, new elements such as copyright transfer agreements were introduced to formalize this transfer. Knowledge in the form of content was converted to a private good as a means of engaging corporate interests to manage the coordination problem for us.

This process stretched over the decades from Maxwell's purchase of Pergamon to the Bayh–Dole Act of 1980, but I would argue that the coincidence of the scaling up of scholarship and scholarly publishing, its ability for the first time to generate a financial surplus and the increasing narratives of knowledge as a private good to be exploited is not accidental. The irony of this is that by creating larger and more clearly defined markets of research audiences, we solved the problem of market scale that troubled early journals (which needed to find both popular and expert audiences to remain commercially viable) by locking wider publics out.

The Internet and the Web also changed everything, but it is not the cost of reproduction that most matters. The critical change for our purpose here is the change in the economics of discovery. As part of our privatization of knowledge we parcelled it up into journals, an industrial broadcast mechanism in which one person aims with as much precision as possible to reach the desired expert audience. Discovering knowledge under the new Web-based regime means that it is no longer necessary to find the expert that knows everything about a subject, but writes for only the selected few. Instead, all one needs is to find the *right* person who just happens to have the *right* piece of knowledge to solve a specific problem.

These two trends—industrial broadcast and bespoke discoverability—are pulling us in opposite directions. The industrial model means creating specialization and labelling. It means the creation of communities and niches that are, for publishers, markets that can be addressed individually. These communities are defined by credentials that validate a deep expertise in a given subject. The ideas of micro-expertise, of a person with no credentials having the key information or insight, radically undermines the traditional dynamics of scholarly group formation. For research communities to embrace the opportunities of the Web, they therefore have to reject, or at least consider as less important, those traditional markers of power and expertise. I do not think it is an accident that those scholarly communities that Michèle Lamont identifies as having the most stable self-conception have a tendency also to be the most traditional in terms of their communication and public engagement.^3^ Lamont identifies history and analytic philosophy in this group. I might add synthetic chemistry as an example from my own experience.

This restructuring of the economics of discovery has profound implications for our understanding of expertise. And it is our cultures of expertise that form the boundaries of our groups—our knowledge clubs—whether they be research groups, disciplines, journals, discussion meetings or scholarly societies. The Web shifts our understanding of public-making. It shifts communication from the need to define and target an expert audience through one-to-many broadcast—a one-to-audience interaction—to a many-to-many environment in which we aim to connect with the right person to discover the right contribution. The importance of the groups remains. The means by which they can, and should, want to communicate has changed radically.

The challenge lies not in giving up on our ideas of expertise, but in identifying how we can create groups that both develop shared understanding that enables effective and efficient communication internally and are also open to external contributions. It is not that defining group boundaries does not matter—it is crucial—but that the shape and porosity of those boundaries need to change. Journals have played a role throughout their history in creating groups, defining boundaries and validating membership. That role remains important. The groups, and their cultures, will need to change to compete and survive.

So we will still have ‘journals’ in 2035 and they will still define and support communities. But the way they do that, and the way they connect with other communities, will almost certainly have radically changed.

## Notes

1 Cameron Neylon, ‘The limits on “Open”: why knowledge is not a public good and what to do about it’, *Science in the open* blog, http://cameronneylon.net/blog/the-limits-on-open-why-knowledge-is-not-a-public-good-and-what-to-do-about-it/ (accessed 7 July 2015); Elinor Ostrom, *Governing the Commons* (Cambridge University Press, 1990).

2 James M. Buchanan, ‘An economic theory of clubs’, *Economica* **32**(125), 1–14 (1965).

3 Michèle Lamont, *How professors think: inside the curious world of academic judgement* (Harvard University Press, Cambridge, MA, 2009).

